# Development of novel *LOXL1* genotyping method and evaluation of *LOXL1*, *APOE* and *MTHFR* polymorphisms in exfoliation syndrome/glaucoma in a Greek population

**Published:** 2013-05-06

**Authors:** Dimitrios Chiras, Konstantina Tzika, Haris Kokotas, Samantha C. Oliveira, Maria Grigoriadou, Anastasia Kastania, Kleanthi Dima, Maria Stefaniotou, Miltiadis Aspiotis, Michael B. Petersen, Christos Kroupis, George Kitsos

**Affiliations:** 1 Department of Ophthalmology, University Hospital of Ioannina, Ioannina, Greece; 2 Department of Ophthalmology, «Aghios Savvas» Hospital, Athens, Greece; 3 Department of Clinical Biochemistry, Attikon University General Hospital, Athens, Greece; 4 Department of Genetics, Institute of Child Health, Athens, Greece; 5 Department of Informatics, Athens University of Economics and Business, Athens, Greece; 6 Department of Clinical Genetics, Aalborg University Hospital, Aalborg, Denmark

## Abstract

**Purpose:**

In the Greek population of Epirus, exfoliation syndrome (XFS) and exfoliation glaucoma (XFG) occur at a high prevalence. In this study, we validate a novel lysyl oxidase-like 1 (*LOXL1*) genotyping method, investigate the previously reported association of *LOXL1* with XFS/XFG, and evaluate apolipoprotein E (*APOE*) and methylenetetrahydrofolate reductase (*MTHFR*) polymorphisms as genetic risk factors for both conditions in our population.

**Methods:**

Blood samples were collected from 82 patients with XFG, 69 patients with XFS, 52 patients with primary open-angle glaucoma (POAG), and 107 controls. *APOE* and *MTHFR* 677C>T genotyping was performed from extracted genomic DNA with established methods. A novel methodology of real-time PCR and melting curve analysis was developed and validated to accurately genotype the *LOXL1* G153D and R141L polymorphisms by using two different fluorescent channels of the LightCycler instrument (Roche) examining each SNP separately.

**Results:**

No significant differences were observed for the *APOE* and *MTHFR* polymorphisms between the patients with XFS, the patients with XFG, and the control subjects. The *APOE* ε2 allele appears to be associated with elevated risk of POAG in our population. Our novel *LOXL1* genotyping method was easy to perform, fast, and accurate. A statistically significant association was found for the *LOXL1* gene with XFS/XFG in this Greek population. The association of XFS and XFG with G153D appeared to be less powerful in this population (XFS: odds ratio [OR]=2.162, p=0.039, XFG: OR=2.794, p=0.002) compared to other populations, and for R141L, the association was proven only with XFG (OR=3.592, p<0.001). Neither of the two *LOXL1* SNPs was significantly associated with POAG.

**Conclusions:**

We confirmed the association between *LOXL1* and XFS/XFG, but the *APOE* and *MTHFR* polymorphisms are not significant risk factors for the development of XFS/XFG in our population of patients from Epirus (Greece).

## Introduction

Exfoliation syndrome (XFS) represents a complex, late onset, generalized disease of the extracellular matrix characterized by the progressive, stable deposition of abnormal fibrillar aggregates in various intraocular and extraocular tissues [1]. The average worldwide prevalence of XFS ranges from 10% to 20% of the general population over the age of 60 years. However, studies have shown much higher prevalence in Nordic and Greek populations [2,3]. In the northwest region of Greece called Epirus, XFS has been diagnosed in 24.3% of the population over the age of 50 years [4]. The underlying causes of the differences in prevalence rates between age-matched geographical and ethnic populations remain unknown but appear to be related to variations in genetic background [5].

XFS is one of the most common causes of glaucoma worldwide [6]. Exfoliation glaucoma (XFG), arising from XFS, may account for 20%–60% of open-angle glaucoma and may even show a higher frequency than primary open-angle glaucoma (POAG) in some populations [7].

Elevated intraocular pressure (IOP) is the most important risk factor for glaucomatous damage. However, pathogenic mechanisms other than elevated IOP thus must be important. Many studies have focused on polymorphisms of several genes as risk factors for glaucoma.

Recently, genetic studies have demonstrated a highly significant association between XFS and sequence variants in the lysyl oxidase-like 1 (*LOXL1*) gene coding for lysyl oxidase-like 1. In a genome-wide association study, Thorleifsson et al. found a strong association between XFS/XFG and three single nucleotide polymorphisms (SNPs) on the *LOXL1* gene in Scandinavian populations [8]. These three risk alleles are the T allele of the non-coding rs2165241 in the first intron, the G allele of dbSNP: rs1048661 (resulting in an arginine to leucine amino-acid change, R141L) and the G allele of rs3825942 (resulting in a glycine to aspartic acid amino-acid change, G135D) in the first exon of the gene. LOXL1, in its carboxy terminal, possesses key oxidative lysine deamination enzymatic activity useful for elastic fiber synthesis and homeostasis, supporting the role of defective elastogenesis and elastosis in the pathophysiology of XFS. Multiple replication studies in non-Scandinavian populations (United States, Australia, Europe, Japan, China, and India) [9], in the Uygur [10] and in a Pakistani population [11] confirmed genetic susceptibility of *LOXL1* polymorphisms to XFS/XFG and verified the *LOXL1* gene as a principal genetic risk factor for this condition worldwide accounting for almost all XFS cases. However, the frequencies of the risk alleles for the *LOXL1* SNPs were also high in the control populations and variable among different ethnic groups [12-17]; therefore, the exact role of the *LOXL1* gene in the pathogenesis of the disease remains to be determined [1].

Methylenetetrahydrofolate reductase (*MTHFR*) and apolipoprotein E (*APOE*) are among the genes that have been investigated in relation to exfoliation and glaucoma [18-21]. Previous studies pointed to a possible association between *APOE* alleles and glaucoma in defined populations [20]. Three common alleles, ε2, ε3, and ε4, code the three major isoforms: Apo E2, E3, and E4, respectively. The ε3 allele is considered the ancestral allele, and ε2 and ε4 are considered variants, based on single point mutations in two amino acid positions: 112 (rs429358) and 158 (rs7412). *APOE* alleles modulate the biologic functions of ApoE in part by altering the binding of the different lipoprotein subclasses [22]. Yilmaz et al. found the *APOE* ε2 allele was significantly associated with the development of XFS in a Turkish population [19].

Previous reports have also indicated that homocysteine is moderately elevated in the aqueous humor, tear fluid, and serum plasma of patients with XFS and XFG [23,24]. The biologic role of hyperhomocysteinemia in glaucoma is not known. However, hyperhomocysteinemia has been linked to vascular disease [25] and has been proposed to contribute to the increased vascular risk observed in patients with XFS, which includes aneurysms of the abdominal aorta [26]. Furthermore, homocysteine causes dysregulation of matrix metalloproteinases and their inhibitors [27], which has been implicated in the pathogenesis of XFG [28]. Reduced activity of a thermolabile form of methylenetetrahydrofolate reductase, caused by a C677T polymorphism in the *MTHFR* gene (rs1801133, alanine to valine amino-acid change), is the most common genetic factor for moderate hyperhomocysteinemia, and a higher prevalence of C677T has been found in patients with POAG [18]. Therefore, this polymorphism is debated as a potential genetic risk factor for XFS, XFG, and POAG.

The primary goal of our study was to develop a novel reliable methodology for detecting the two nonsynonymous coding *LOXL1* SNPs, G153D and R141L. Thus far, all previous studies used DNA sequencing for detecting these two *LOXL1* SNPs. However, the studies overlooked the fact that the first exon is highly homologous with the other four paralogous genes *LOXL*, *LOXL2*, *LOXL3*, and *LOXL4*. In this context, we performed a bioinformatics study to design a highly specific assay for our gene of interest with real-time PCR and melting curve analysis. The platform used was the LightCycler engine. Then we applied this validated methodology along with already published assays for *APOE* and *MTHFR* genotyping in a significant number of patients with XFS and XFG and corresponding controls originating from the Greek Epirus area.

## Methods

### Patients and clinical criteria

Blood samples were collected from 82 patients with XFG (mean age±standard deviation [SD] 74.5±7.7 years, 55.6% male), 69 patients with XFS (76.2±7.2 years, 39.1% male), 52 patients with POAG (69.4±8.8 years, 51% male), and 107 controls without exfoliation or glaucoma (74.4±8.2 years, 43.9% male). The patients and controls were matched for age and gender, and all were from the same geographic area in northwest Greece (Epirus). All participants were monitored at the Department of Ophthalmology, University Hospital of Ioannina and signed written informed consent before enrolling. The study was conducted in accordance with the Declaration of Helsinki and subsequent revisions. The protocol was approved by the Ethics Committees of the University Hospital of Ioannina and the Institute of Child Health, Athens. All subjects and controls underwent a comprehensive ocular examination including vision and refraction, slit-lamp biomicroscopy, Goldmann applanation tonometry, gonioscopy, dilation of the pupils for examination of the lens (for the presence of exfoliation deposits on the anterior lens capsule), funduscopy, and repeated Humphrey 24-2 static threshold perimetry. Ocular and medical history was also recorded.

XFS was defined by the presence of typical exfoliation material on the anterior lens capsule and/or at the pupillary margin in one or both eyes with a normal optic disc and visual field and IOP ≤21 mmHg in both eyes. Inclusion criteria for patients with POAG were untreated IOP ≥22 mmHg on at least two diurnal curves, open-normal angle, glaucomatous optic disc, and at least three Humphrey visual field tests with glaucomatous defects. Patients with secondary or closed-angle glaucoma were excluded. The criteria for the diagnosis of POAG followed the guidelines of the European Glaucoma Society [29]. In addition to the signs of open-angle glaucoma, XFG was defined by the presence of characteristic slit-lamp findings including exfoliation deposits on the anterior lens capsule and/or at the pupillary margin in one or both eyes. Controls were individuals of similar age as the patients without any evidence of exfoliation deposits on intraocular tissues. The controls’ IOPs were in the normal range (≤21 mmHg) with normal-appearing optic nerves, open-angle, normal Humphrey visual field tests, and no family history of glaucoma.

### DNA isolation and *APOE* and *MTHFR* 677C>T genotyping

Genomic DNA was extracted using a salting-out procedure from nucleated cells from the venous EDTA blood samples of all 306 subjects [30]. Genotyping of polymorphic *APOE* alleles was performed after PCR amplification using forward primer APOE-F: 5′-TCC AAG GAG CTG CAG GCG GCG CA-3′ and reverse primer APOE-R: 5′-ACA GAA TTC GCC CCG GCC TGG TAC ACT GCC A-3′ (protocol included initial denaturation at 94 °C for 5 min followed by 40 cycles of denaturation at 94 °C for 30 s, annealing at 65 °C for 30 s, extension at 70 °C for 1.5 min, and final extension at 70 °C for 10 min). The 227 bp PCR product was digested with HhaI (New England Biolabs, Beverly, MA) into 91, 81, 72, and 48 bp fragments [31,32].

Detection of the *MTHFR* 677C>T polymorphism was performed with PCR amplification using forward primer MTHFR1: 5′-TGA AGG AGA AGG TGT CTG CGG GA-3′ and reverse primer MTHFR2: 5′-AGG ACG GTG CGG TGA GAG TG-3′. The PCR was performed according to the following protocol: 95 °C for 3 min, followed by 30 cycles of 94 °C for 1 min, 58 °C for 1 min, 72 °C for 2 min, and final extension of 72 °C for 8 min. The products were subsequently digested with HinfI (New England Biolabs). The 677C>T substitution creates a HinfI recognition sequence that digests the 198 bp PCR fragment into 175 and 23 bp fragments [33,34]. For both methods, all PCR products were digested at 37 °C for 24 h, separated by electrophoresis on 3% NuSieve – 1% agarose gels, and visualized with ethidium bromide staining.

### *LOXL1* novel methodology

We selected the real-time PCR LightCycler instrument (Roche Applied Science, Basel, Switzerland) to develop the novel *LOXL1* methodology for the G153D and R141L polymorphisms. This instrument uses the format of dual hybridization probes (anchor and sensor probes labeled with fluorescein [FL] and LC640 or LC705 dyes) that provide additional specificity. Only when both anchor and sensor probes locate their specific targets within the PCR product does fluorescence resonance energy transfer (FRET) emission occur and can then be continuously monitored by the instrument in the F2 (for LC640) or the F3 (for LC705) channel (if two different SNPs are assessed). Allele discrimination is achieved with melting curve analysis of the PCR product after the cycling ends.

**Figure 1:**
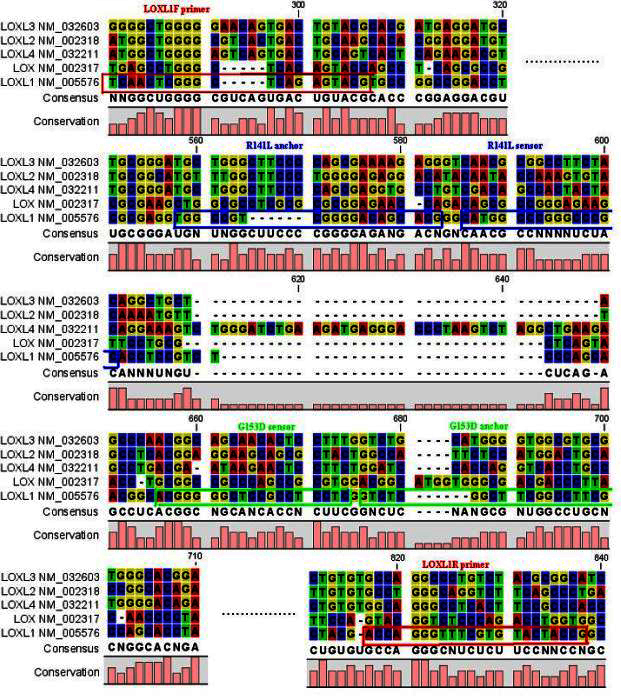
Partial alignment of lysyl oxidase-like 1, lysyl oxidase-like 2, lysyl oxidase-like 3, and lysyl oxidase-like 4 genes by CLC Sequence Workbench. Sequences of lysyl oxidase-like 1 (*LOXL1*) primers are boxed in a red frame, probes for the R141L single nucleotide polymorphism (SNP) in blue, and probes for the G153D SNP in green (only these areas are shown).

CLC-Sequence Workbench software (CLC Bio, Aarhus, Denmark) was used to align the GenBank-imported DNA sequences of the *LOXL*, *LOXL1*, *LOXL2*, *LOXL3*, and *LOXL4* genes (Figure 1), and the tool proved helpful in designing the primers and probes (unique for our gene of interest) because these paralogous genes are significantly homologous in exon 1. This concept was used successfully in an analogous situation in a previous study by our group for the Fc fragment of IgG, low affinity IIa (*FCGR2A*) gene [35]. The sequences of the primers and probes for the proposed real-time PCR assay for the rs3825942 (G153D, c.458G→A) and rs1048661 (R141L, c.422G→T) SNPs were designed in silico by our team and synthesized by TIB MolBiol (Berlin, Germany). The sequences are shown in Table 1. Probes were 100% complementary with the rare A and T alleles, correspondingly, for G153D and R141L. Because these two SNPs are close in the *LOXL1* exon 1 sequence and their corresponding sensor probes are labeled with different but compatible dyes, after developing the two separate assays, we also attempted to develop a combined method by using all four probes in the same reaction. We wanted to screen with a cost-effective combined assay and then repeat a separate SNP assay only in cases of doubt in the melting curve analysis.

**Table 1:** Sequences of *LOXL1* primers and probes used for either the separate or the combined assays developed for the detection of R141L and G153D SNPs.

Name	Oligonucleotide sequence	Tm (°C)	GenBank genomic location NG_011466
Common Forward primer, LOXL1F	TCAACTCGGGCTCAGAGTACG	59,0	501–521
Common Reverse primer, LOXL1R	CGGTAGTACACGAAACCCTGGT	59,9	933–954
R141L Anchor	5′-TGGCCGTCGGGGACAGCACG-FL*	72,2	729–748
Sensor probes	LC705-CATGGCCCTGGCCCGC- 3′	64,9	789–807
G153D sensor	5′-ACGGGGACTCCGCCTCCTC-FL*	66,0	751–766
Anchor probes	LC640-GTCTCGGCTTCGGCCTTCGCCAG-3′	73,2	809–831

Probes were 100% complementary with the rare A and T alleles correspondingly (shown underlined). *FL=fluorescein label

We used a 1:4 primer ratio to perform asymmetric real-time PCR since this ratio produces a more single-stranded template for the probes to bind [35]. The amplification reaction occurred in glass capillaries (Roche Applied Science), and the 10 μl total volume mixture in the case of single SNP detection consisted of 1 μl genomic DNA as the template (100 ng/μl), 0.2 μl forward primer *LOXL1F* 20 pmol/μl, 0.8 μl reverse primer *LOXL1R* 20 pmol/μl (1:4 ratio of the two primers), 0.6 μl FL-probe 3 mM, 0.6 LC-probe 3 mM (either LC640 or LC705), 0.6 μl molecular-grade dimethyl sulfoxide (due to high GC context of this *LOXL1* area), 1.6 μl MgCl_2_ 25 mM (final optimal 4 mM concentration), 1 μl of FastStart DNA MasterPlus Mix 10X (Roche Applied Science), and H_2_O to supplement up to 10 μl. After preincubation at 95 °C for 10 min for hot-start activation of the polymerase enzyme, the three-step cycling protocol included 45 cycles of denaturation at 95 °C for 20 s, annealing at 60 °C for 20 s, and extension at 72 °C for 20 s. The temperature ramp rate was 20 °C/s. Emitted fluorescence was measured at the end of each annealing step at either the F2 (for the LC640 probe) or the F3 channel (for the LC705 probe). After amplification, the melting curve analysis was started by raising the temperature at 95 °C for 30 s and then at 45 °C for 60 s, proceeded with a slow heating step up to 85 °C at a ramp rate of 0.3 °C/sec during which fluorescent measurements were continuously collected, and ended with a final cooling step at 40 °C for 30 s.

### DNA sequencing

After the real-time PCR and melting curve analysis were performed, glass capillaries for selected samples were inverted and spun in Eppendorf tubes. About 7.5 μl from the collected PCR reaction product (454 bp, size was also checked with agarose gel electrophoresis) was incubated with 3 μl ExoSAP-IT (USB Affymetrix, Santa Clara, CA). Then 5 μl of the treated PCR product was used as a template for the cycle sequencing reaction with the Big Dye Terminator v1.1 kit (Applied Biosystems, Life Technologies, Foster City, CA). Samples were ethanol-precipitated and run in a 47-cm capillary filled with POP-6 polymer in an ABI 310 Genetic Analyzer (Applied Biosystems) and manually base-called with the Chromas Lite 2.01 software (Technelysium Pty Ltd, South Brisbane, Australia).

### Statistical analysis

Statistical analysis was performed using the SPSS 19.0 software (IBM-SPSS Inc., Chicago, IL). The criterion used for statistical significance was p<0.05. Normality of distribution of continuous variables was performed with the Kolmogorov–Smirnov test and a mean value comparison with analysis of variance (ANOVA). Descriptive statistics were used to calculate the frequencies and percentage of the categorical variables. Proportions of groups were compared with the χ^2^ test. Either Fisher’s exact test or the Pearson chi-square test [36] was used to compare the patient and control groups for possible associations between SNP allele frequency/haplotype frequency and disease state. The odds ratios (ORs) and 95% confidence intervals (95% CIs) were calculated. Step-wise logistic regression analysis was used to evaluate multiple covariate effects when the combined XFS and XFG group was compared in relation to the control subjects. The covariates were all genes (two *LOXL1* SNPs, *APOE*, *MTHFR*), sex, age of examination, diabetes, hypertension, cardiovascular disease, and vascular disease. Checking of conformance with Hardy–Weinberg equilibrium (HWE) was done by using the SNPstats software [37]. Individual haplotypes and their estimated population frequencies were also inferred by using SNPstats. Power analysis was conducted using G*Power 3.1.5 [38]. For all, Pearson chi-square power calculations were computed with effect size=0.03 – medium.

## Results

### *LOXL1* genotyping method development and validation

**Figure 2:**
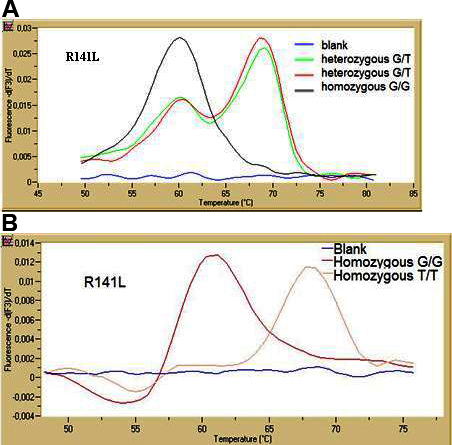
Melting curve analysis for R141L single nucleotide polymorphism in channel F3 (for LC705 detection). **A**: Two peaks are shown for the two heterozygous G/T samples and one single peak for the homozygous G/G. **B**: Two peaks are shown for the homozygous T/T sample and one single peak for the homozygous G/G.

**Figure 3:**
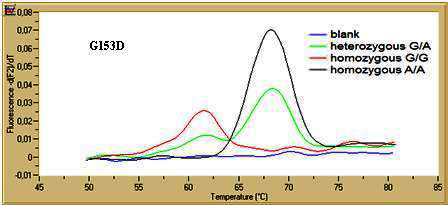
Melting curve analysis for G153D single nucleotide polymorphism in channel F2 (for LC640 detection), for one heterozygous G/A, one homozygous G/G, and one homozygous A/A.

Our novel methodology was easy to perform, fast (within 40 min after DNA isolation), and reliable in the format of separate SNP assays. The method was accurate when compared to the method used most often as the gold standard in genetic analysis: DNA sequencing (n=40, 100% concordance, data not provided). Amplification demonstrated good efficiency (E=1.86) and reproducibility (coefficient of variation, CV, or Cq between-run precision <2.5%). The characteristic melting curves for each SNP assay are shown in Figure 2A,B and Figure 3. Peaks for the two alleles were clearly separated in the melting curve analysis of both assays (ΔTm>6 °C). The melting points (Tm) for both rare alleles A and T were higher than the corresponding G risk alleles as expected because the probes were 100% complementary with the rare alleles. For G153D, the Tm was 68.22 °C for the A allele and 61.91 °C for the G allele (ΔTm=6.31 °C). For R141L, the Tm was 68.63 °C for the T allele and 61.28 °C for the G allele (ΔTm=7.35 °C). Reproducibility in the melting curve characteristics was satisfactory (CV of each Tm<2.5%). Results from the combined assays for the two SNPs were not as straightforward due to artifacts in the dual heterozygous samples. In addition, although this approach is cost-effective, many repeats had to be performed, and finally, it was not implemented.

### *LOXL1* results

We obtained *LOXL1* genotyping results from 71 patients with XFG (mean age ± SD, 74.7±7.4 years, 52.9% males), 54 patients with XFS (76.0±6.8 years, 35.2% males), 43 patients with POAG (69.2±9.0 years, 54.8% males), and 97 controls without exfoliation or glaucoma (74.8±7.5 years, 43% males; a few DNAs did not amplify or were limited in amount). Age and sex did not differ significantly between the groups (ANOVA, χ^2^). The distributions of allele and genotype frequencies for both *LOXL1* SNPs are shown in Table 2. All frequencies were in HWE.

**Table 2:** Distribution of *LOXL1* sequence variants in XFG, XFS, POAG patients and controls.

G153D	n	Alleles		P value	Genotypes			P value†	OR† (95%CI)
		A	G		AA	GA	GG		
XFG	71	19	123	0.002*	0	19	52	0.002*	2.794
		0.13	0.87		0	0.27	0.73		(1.445–5.401)
XFS	53	17	89	0.032*	0	17	36	0.039*	2.162
		0.16	0.84		0	0.32	0.68		(1.073–4.357)
POAG	43	16	70	>0.05	0	16	27	>0.05	
		0.19	0.81		0	0.37	0.63		
Controls	97	53	141		4	45	48		
		0.27	0.73		0.04	0.46	0.49		
R141L	n	T	G	P value	TT	GT	GG	P value†	OR† (95%CI)
XFG	70	15	125	<0.001*	1	13	56	<0.001*	3.592
		0.11	0.89		0.01	0.19	0.8		(1.760–7.329)
XFS	54	23	85	0.399	2	19	33	0.39	1.411
		0.21	0.79		0.04	0.35	0.61		(0.713–2.791)
POAG	43	24	62	>0.05	4	16	23	>0.05	
		0.28	0.72		0.09	0.37	0.53		
Controls	93	49	137		5	39	49		
		0.26	0.74		0.05	0.42	0.53		

P values and OR are calculated when compared to controls. Asterisks (*) denote statistical significance. The daggers (†) indicate that the p values and OR ratios are derived from the comparison of the specific risk GG genotype versus all of the others.

The risk G allele of the G153D SNP was found in a significantly higher frequency in the patients with XFS (84%; p=0.032) and XFG (87%; p=0.002) compared to the control samples (73%). The 153 GG homozygous genotype versus GA+AA (recessive mode of inheritance) was also significantly associated with XFS (68%; p=0.039; OR=2.162, 95% CI=1.073–4.357; G*Power=0.95) and XFG (73%; p=0.002; OR=2.794, 95% CI=1.445–5.401; G*Power=0.97) compared to controls (49%). In addition, the risk G allele of the R141L SNP was significantly more frequent in XFG (89%; p<0.001) than in the controls (74%) but not in the patients with XFS. The 141 GG homozygous genotype versus GT+TT was also significantly associated with XFG (p<0.001; OR=3.592, 95% CI=1.760–7.329; G*Power=0.96) but not with XFS (p=0.39; G*Power=0.95). We did not find a highly significant association between the *LOXL1* SNPs and POAG (p>0.05; G*Power>0.93). The association of XFS and XFG with G153D appeared to be less powerful in our population (XFS: OR=2.162, p=0.039, XFG: OR=2.794, p=0.002) than in Nordic populations. For R141L, the association was proved only with XFG (OR=3.592, p<0.001). In addition, we compared the allelic and genotypic frequencies of the two *LOXL1* SNPs between the patients with XFS and XFG and found significant differences for R141L. The G allele of R141L was found in a significantly higher frequency in XFG (89%; p=0.032) compared to XFS (79%). The 141 GG genotype was also significantly associated with XFG (80%; p=0.027; OR=2.545, 95% CI=1.142–5.673; G*Power=0.91) compared to XFS (61%).

Haplotype analysis of the two *LOXL1* SNPs estimated that only three of the four possible haplotypes were detected in our samples (D’=1); the haplotype formed by the two protective alleles (TA) was not detected. However, the estimated high-risk GG haplotype frequency in apparent linkage disequilibrium in our population was lower than in Nordic populations (in Greece: 75.8% in XFG, 62.5% in XFS, 46.1% in controls while in Iceland: 81.4% in XFG, 49.8% in controls; and in Sweden: 83.3% in XFG, 56.1% in controls) [8].

In our step-wise logistic regression multivariate analysis, after adjusting for the effect of all genes evaluated (including *MTHFR* and *APOE*) and factors known to influence the prevalence of XFS/XFG, including sex, diabetes, hypertension, cardiovascular disease, and vascular disease, both SNPs remained significantly associated with the risk of developing XFS and XFG. G153D, R141L, and hypertension showed an association with XFS risk (OR=6.122, 95% CI=2.705–13.859, p<0.001; OR=3.896, 95% CI=1.715–8.853, p=0.001; OR=2.289, 95% CI=1.142–4.591, p=0.02, respectively). G153D and R141L showed also an association with XFG risk (OR=3.384, 95% CI=1.645–6.958, p=0.001 and OR=4.231, 95% CI=1.964–9.117, p<0.001, respectively).

Finally, the two SNPs were analyzed for their ability to predict affection status as a general population genetic test for XFG. The R141L demonstrated 98.6% clinical sensitivity (69 of 70 cases have the G allele) but only 5.4% clinical specificity (five of 93 controls lack the G allele) as a diagnostic test for XFG. G153D demonstrated 100% sensitivity (71 of 71 cases have the G allele) but only 4.1% specificity (four of 97 controls lack the G allele).

### *MTHFR* and *APOE* results

No significant differences in either the genotype distribution or allele frequencies of the *MTHFR* 677C>T polymorphism were found between the patients with POAG, XFS, and XFG and the control subjects (p>0.05; G*Power>0.96). Furthermore, the homozygous TT genotype was more frequently observed in the cohort of the control subjects (20%) than in the population of the patients from Epirus (13%, 13%, and 15% for the patients with POAG, XFS, and XFG, respectively).

We compared the *APOE* genotypes containing at least one ε2 allele (ε2/ε2, ε2/ε3, ε2/ε4: T in codon 158) and the *APOE* genotypes without an ε2 allele (ε3/ε3, ε4/ε4, ε3/ε4: C in codon 158) between the patients with POAG, XFS, and XFG and the control subjects. The T codon 158 genotype was found in 5% of the control subjects versus 19%, 12%, and 10% of the patients with POAG, XFS, and XFG, respectively. We did not find any significant differences in the *APOE* ε2 carriers and non-ε2 carriers between our population of patients with XFS or XFG and the cohort of control subjects (p>0.05; G*Power>0.95). The only significant difference was the higher frequency of the ε2 allele in the patients with POAG (19%) relative to the control group (5%, p=0.012), and it was associated with an increased risk of developing POAG (OR=4.35, 95% CI=1.28–14.72; G*Power=0.92).

## Discussion

XFS is a complex, multifactorial, late onset disease with incidences increasing with age and high prevalence in Greek patients from the region of Epirus [4]. Since XFS is a late onset disease, this study included subjects aged 60 years or older as controls.

XFS and XFG have been shown to demonstrate familial aggregation. However, a simple inheritance pattern is not evident, suggesting a complex inheritance model caused by the contributions of multiple genes and/or environmental influences. To identify genetic risk factors for XFS and XFG, genetic analyses of several candidate genes have been performed but have often achieved conflicting results among the various ethnic populations that were studied.

We confirmed the association between *LOXL1* and XFS and XFG in a significant number of well-ascertained patients originating from Epirus, Greece (all examined by the same experienced ophthalmologist specializing in glaucoma, Prof. G. Kitsos). The association in our study is not as strong as that seen in Scandinavia [8]. Moreover, although the G153D was associated with XFS and XFG, interestingly, the second SNP showing significance in the Nordic population, R141L, is not significantly associated with XFS but only with XFG in our population (and in a stronger fashion than for G153D). In the present study, the allelic and genotypic frequencies of R141L in patients with XFS and XFG were compared, and significant differences were demonstrated between these two groups, which have not been reported so far. This SNP has been linked to altered *LOXL1* expression levels with the risk GG genotype showing reduced levels [39]. In certain XFS ocular tissues, *LOXL1* expression is initially upregulated but in later stages is reduced and more markedly in XFG tissues [39,40]. Apparently, the TT genotype can assist in sustaining important levels of *LOXL1* activity and therefore act in a protective fashion. However, the other tested SNP does not seem to affect *LOXL1* expression or enzymatic activity (the catalytic center resides in the final part of the protein) [39]. This SNP could affect substrate targeting and binding or could be a tag-SNP for another nearby locus association. Based our results, we suggest that the *LOXL1* gene may contribute independently to the onset of XFG rather than through IOP elevation and subsequent glaucoma. In addition, the Greek population from Epirus has a similar prevalence of XFS/XFG when compared to the Nordic populations; however, different distributions of *LOXL1* genotypes between the two populations exist, indicating that additional factors may influence the development of XFS/XFG.

The SNPs of *LOXL1* did not exhibit any significant association with POAG similar to previous studies of European, American, and other populations suggesting that different genetic factors contribute to this condition [9,41,42]. The risk for XFG in the Greek population of Epirus was associated most strongly with the GG haplotype. However, the frequency of this high-risk haplotype in our population was lower than in Nordic populations.

We demonstrate that the association of the two SNPs with XFG is not strong enough to justify a general population diagnostic test for the disease. In our study, although the two SNPs individually have high clinical sensitivity, their clinical specificity is poor (4.1% to 5.4%) due to their high prevalence in the control subjects, which is in agreement with previous studies [43,44]. This statement does not therefore justify their inclusion in the microarray panels used by the direct-to-consumer (DTC) laboratories for screening of the general population. Screening of the XFS population with R141L could potentially assist in isolating individuals who would eventually develop XFG. This could be proved with large prospective studies. The high prevalence of *LOXL1* variants in the control subjects and patients with XFS suggests that there may be other protective genes or environmental factors that could provide influence by delaying the development of XFS/XFG.

Studies have suggested that hyperhomocysteinemia may be associated with XFS/XFG or POAG. We found no correlation between the C677T *MTHFR* polymorphism and XFS/XFG and POAG, which is in accordance with several previous studies, which strongly suggests that this polymorphism itself is not a major risk factor for the investigated diseases [45,46]. In our control population, the prevalence of the TT genotype was found in 20% of the individuals, a prevalence significantly higher than that reported in Northern European populations (5%–10%). In European populations, the prevalence of homozygosity for the *MTHFR* 677T allele has been reported to be between 4.0% and 26.4% with increasing prevalence in Southern Europe [47]. We confirm the variable frequency of homozygotes for the 677TT in populations from different geographic areas, which seems to have an increasing north-to-south cline. Failure to confirm an association between the C677T variant and XFS/XFG or POAG may be due to differences between the patient populations of the studies in terms of ethnic background, phenotypic stratification, matching of controls, or the effect (sample) size used to conduct the power analysis.

The results of this study suggest that *APOE* alleles may influence the risk of POAG but have no effect on the susceptibility of XFS/XFG. We did not find evidence of a significant association between the *APOE* ε2 allele and XFS found in positive association with the disease in the study by Yilmaz et al. [19]. Based on our results, we suggest that *APOE* alleles cannot be considered principal risk factors for the development of XFS or XFG, which is in agreement with previous studies [21]. In our population, the ε2 allele appears to be associated with elevated risk of POAG. The *APOE* ε2 allele was associated with elevated IOP by Junemann et al. [48]. Earlier studies evaluated the possible association between *APOE* alleles and POAG. However, the results of these studies are contradictory [20]. A possible reason for these dissimilar findings could be regional differences in the *APOE* allele frequencies; the association between *APOE* and POAG may vary among ethnic groups [19]. *APOE* might have a more obvious effect in populations exposed to different environmental factors such as diet and hormone levels or with a different genetic background. Further investigations, by us and others, involving a larger number of patients and considering this complex interplay between genetic and environmental factors are warranted to fully appreciate the role of ApoE in XFS/XFG and POAG.

Because our study mainly evaluated SNPs in a medium-size population, we report the power calculations for all Pearson chi-square tests examining genotype associations with XFS, XFG, and POAG [36]. The effect size used for the power analysis of the Pearson chi-square tests represents the magnitude of the effect and is either known from previous research or specified by the researcher. Conventional values for size effects have been suggested by Cohen [49,50]. We used the suggested medium effect size, and the post hoc achieved power was presented with the statistical significance level set at 0.05.

The method developed means advancement in genotyping because this method improves specificity in the assay by adding the two probes and the well-designed primers. The PCR-based DNA sequencing method cannot always accurately discriminate highly homologous sequences, and genotyping errors can occur even rarely (not seen in our limited data set used for comparing methods) but at a significant percentage that could affect genetic associations. This type of error in homologous genomic areas is expected to rise with the advent of next-generation sequencing methods in which reactions occur in much smaller fragments. The method is rapid, accurate, reproducible, amenable to automation, and higher throughputs. In addition, the method is less prone to contamination because amplification and genotyping occur in a closed tube format. This method could be extremely cost-efficient if the genotyping assays could be combined; however, this could lead to artifacts and is not suggested. The single genotyping format is preferred.

This is the first study to show that the polymorphisms of *LOXL1* are associated with XFS/XFG in Greek patients. Although a major risk genetic factor for XFS/XFG has been identified in *LOXL1*, genetic testing based on *LOXL1* for this disorder is problematic. In our population, we suggest that the R141L allele of *LOXL1* may contribute to XFG onset independently rather than through IOP elevation and subsequent glaucoma. In conclusion, other genes must be involved with this complex and late onset disorder. Further studies involving an even larger number of patients are needed to identify these genes or factors interacting with the *LOXL1* gene, as well as other genes that may contribute through other mechanisms to the risk of developing XFS/XFG. We propose that not only the entire *LOXL1* gene—including intronic and promoter sequences—but also other *LOX* family member genes (*LOX*, *LOXL2*, *LOXL3*, and *LOXL4*) whose protein products are also expressed in ocular tissues could be strong candidates and might be associated with XFS/XFG. In this context, analogous assays such as ours are needed for discrimination between these highly homologous genomic areas. This line of research may eventually help elucidate some of the basic mechanisms behind glaucoma development in general and provide diagnostic and/or prognostic laboratory tools for clinicians and patients.
